# Inhibition of Collagen-Induced Platelet Aggregation by the Secobutanolide Secolincomolide A from *Lindera obtusiloba* Blume

**DOI:** 10.3389/fphar.2017.00560

**Published:** 2017-08-22

**Authors:** Sang-Hyuk Jung, Joo-Hui Han, Hyun-Soo Park, Jung-Jin Lee, Seo Young Yang, Young Ho Kim, Kyung-Sun Heo, Chang-Seon Myung

**Affiliations:** ^1^Department of Pharmacology, College of Pharmacy, Chungnam National University Daejeon, South Korea; ^2^Korean Medicine Application Center, Korea Institute of Oriental Medicine Daegu, South Korea; ^3^Department of Natural Product Chemistry, College of Pharmacy, Chungnam National University Daejeon, South Korea; ^4^Institute of Drug Research and Development, Chungnam National University Daejeon, South Korea

**Keywords:** *Lindera obtusiloba*, secolincomolide A, antiplatelet action, glycoprotein VI receptor, cyclooxygenase-1 metabolites

## Abstract

Atherothrombosis is one of the main underlying cause of cardiovascular diseases. In addition to treating atherothrombosis with antithrombotic agents, there is growing interest in the role of natural food products and biologically active ingredients for the prevention and treatment of cardiovascular diseases. This study aimed to investigate the effect of secolincomolide A (**3**) isolated from *Lindera obtusiloba* Blume on platelet activity and identify possible signaling pathways. In our study, the antiplatelet activities of **3** were measured by collagen-induced platelet aggregation and serotonin secretion in freshly isolated rabbit platelets. Interestingly, **3** effectively inhibited the collagen-induced platelet aggregation and serotonin secretion via decreased production of diacylglycerol, arachidonic acid, and cyclooxygenase-mediated metabolites such as thromboxane B_2_ (TXB_2_), and prostaglandin D_2_ (PGD_2_). In accordance with the antiplatelet activities, **3** prolonged bleeding time and attenuated FeCl_3_-induced thrombus formation in arterial thrombosis model. Notably, **3** abolished the phosphorylation of phospholipase Cγ2 (PLCγ2), spleen tyrosine kinase (Syk), p47, extracellular signal-regulated kinase 1/2 (ERK1/2), protein kinase B (Akt) by inhibiting the activation of the collagen receptor, glycoprotein VI (GPVI). Taken together, our results indicate the therapeutic potential of **3** in antiplatelet action through inhibition of the GPVI-mediated signaling pathway and the COX-1-mediated AA metabolic pathways.

## Introduction

Platelets play crucial roles in physiological hemostatic and pathological thrombosis, leading to events of coronary heart disease and its associated complications (Stalker et al., 2012). Upon blood vessels damaged, adhesive micromolecules and soluble platelet agonists, such as collagen, von Willebrand factor, adenosine diphosphate (ADP), and thrombin, are exposed or locally generated at the injury site (Steinhubl and Moliterno, 2005; Fogelson and Neeves, 2015). These molecules initiate platelet adhesion, activation, and aggregation to induce further thrombus formation.

Of the adhesive ligands, collagen fibers are highly thrombogenic, and the platelet glycoprotein VI (GPVI) receptor-mediated signaling pathway predominantly responds to collagen-induced platelet activation (Jandrot-Perrus et al., 2000). This signaling pathway involves spleen tyrosine kinase (Syk) and phospholipase C-γ2 (PLCγ2), and leads to thromboxane A_2_ (TXA_2_) synthesis and granule secretion. Collagen-induced platelet activation changes platelet shape and the release of thrombotic substances that recruits activated platelets to the developing thrombus (Moog et al., 2001). Following the activation of platelets by collagen, arachidonic acid (AA) levels are markedly increased via phospholipase A_2_ (PLA_2_)-mediated phospholipid hydrolysis (Turini and Holub, 1993). In platelets, cyclooxygenase (COX) and lipoxygenase (LOX) are two major enzyme families that catalyze the formation of prostaglandins and the leukotrienes, respectively (Porro et al., 2014). Prostaglandins include prostaglandin D_2_ (PGD_2_), thromboxane A_2_ (TXA_2_), and prostacyclines, and leukotrienes include cysteinyl leukotrienes and 12-hydroxyeicosatetraenoic acid (12-HETE) (Porro et al., 2014). Both COX and LOX metabolites are key mediators of vascular inflammation and atherothrombosis associated with platelet activation. Therefore, important role of TXA_2_ and 12-HETE have been suggested as pro-aggregatory factors in platelet function (Lee et al., 2014; Maskrey et al., 2014).

*Lindera obtusiloba* (*L. obtusiloba*) Blume is a small tree or shrub widely distributed across the Korea peninsula, China, and Japan (Yook, 1989). Crude extracts of *L. obtusiloba* have shown to increase the antioxidant effect on the HepG2 cell *in vitro* and inhibit hepatotoxicity *in vivo* (Hong et al., 2013b). Neolignan, lignans and phenolic glycosides isolated from the stems of *L. obtusiloba* Blume were reported to have an inhibitory effect on mast-cell-derived allergic inflammation by inhibiting histamine release and suppressing the gene expressions of pro-inflammatory cytokines (Choi et al., 2013, 2014). Recently, *L. obtusiloba* leaf extract showed an ability to inhibit various agonists-induced platelet aggregation and collagen-induced TXA_2_ production in rat platelets, having antiplatelet and antithrombotic effects (Kim et al., 2016). However, the active ingredients in *L. obtusiloba* responsible for antiplatelet and antithrombotic effects and its underlying molecular mechanisms are still unknown.

Here we report, for the first time, the crucial role of secolincomolide A (**3**, see in Supplementary Figure 1 and Materials and Methods section), which is isolated from chloroform fraction of *L. obtusiloba* extract, in down-regulation of platelet activation, aggregation, and aggregatory metabolite formation in collagen-treated platelets through inhibition of the GPVI signaling pathway. Antiplatelet action of **3** was confirmed by examining bleeding time and thrombus formation *in vivo*.

## Materials and Methods

### Plant Material

The stems of *L. obtusiloba* (Supplementary Figure 1) were purchased at Cyber kyungdong market, Seoul, South Korea, in May 2010 and were taxonomically identified by one of authors (Prof. Young Ho Kim). A voucher specimen (CNU10109) was deposited at the Herbarium of College of Pharmacy, Chungnam National University, Daejeon, South Korea.

### Extraction and Isolation

A methanol (MeOH) extract (185.3 g) of *L. obtusiloba* stems was suspended in water and partitioned with chloroform (CHCl_3_, 52.4 g), ethyl acetate (EtOAc, 30.4 g), and butanol (*n*-BuOH, 45.2 g), successively. The CHCl_3_ extract was subjected to various column chromatography procedures, and four compounds were isolated. Structures of known compounds (**1**–**4**) were elucidated by comparing spectroscopic data to published values (Bussey et al., 2014) and identified as asarinin (**1**; 124.9 mg) (Navarrete et al., 2003; Han et al., 2015b), secoisolitsealiicolide B (**2**; 49.9 mg) (Cheng et al., 2010), secolincomolide A (**3**; 34.1 mg) (Tsai et al., 2002), and secomahubanolide (**4**; 22.4 mg) (Cheng et al., 2005) (Supplementary Figures 2, 3).

### Washed Rabbit Platelet Preparation and Platelet Aggregation Assay

Male New Zealand white rabbits were purchased from Sam-Tako Animals (Osan, South Korea). The rabbits were acclimatized for 1 week at 24°C with 55% humidity and allowed free access to a standard commercial pellet (Wonju, South Korea) and drinking water. All animal handling performed according to the instructions of the Committee for Ethical Usage of Experimental Animals in Chungnam National University. The protocol regarding the procedure of blood collection from the ear artery of rabbits was approved by the Committee (CNU-00731).

Blood was collected from the ear artery of white rabbits directly into an anticoagulant citrate dextrose (ACD) solution containing 2.2% trisodium citrate, 0.8% citric acid, and 2% dextrose (w/v). This procedure yielded a 1:0.15 (v/v) blood/ACD mixture. Washed platelets were prepared as described previously (Lee et al., 2009). Briefly, platelet rich plasma (PRP) was obtained by centrifugation of rabbit blood at 1,100 rpm for 10 min. The platelets were sedimented by centrifugation at 3,000 rpm for 10 min and washed with Hepes buffer 1 (137 mM NaCl, 2.7 mM KCl, 1 mM MgCl_2_, 5.6 mM glucose, and 3.8 mM Hepes, pH 6.5) containing 0.35% bovine serum albumin (BSA) and 0.4 mM EGTA. The washed platelets were adjusted to a concentration of 4 × 10^8^ cells/mL in Hepes buffer 2 (pH 7.4, Hepes buffer 1 without EGTA).

Platelet aggregation was measured by the turbidimetry method using a Chrono-log platelet aggregometer (Havertown, PA, United States) (Born and Cross, 1963; Lee et al., 2014). Briefly, the washed platelet suspension was incubated at 37°C in the aggregometer with stirring at 1,000 rpm followed by treatment of dimethyl sulfoxide (DMSO) as a vehicle, various *L. obtusiloba* extracts or **3**. After a 3 min preincubation with the extracts or **3**, platelet aggregation was induced by addition of collagen (5 μg/mL), AA (100 μM), thrombin (0.1 U/mL) or convulxin (500 ng/mL).

### Flow Cytometry Assay

Washed rabbit platelets were incubated with either DMSO or **3** for 15 min and then stimulated with a collagen, for 5 min. The activated cells were fixed with 2% formalin in phosphate buffered saline (PBS) for 1 h followed by incubation with FITC-conjugated GPVI antibody or control mouse IgG for 2 h at room temperature. After washing, the surface expression of GPVI was analyzed using a FACS Calibur flow cytometer (BD Biosciences).

### Serotonin Secretion Assay

Serotonin release was determined as described previously (Lee et al., 2009). In brief, washed rabbit platelet suspension was treated with 5 μM imipramine as a serotonin reuptake inhibitor to prevent reuptake of secreted serotonin. Then, cells were treated with various concentrations of **3** or DMSO at 37°C for 3 min prior to the addition of agonist, such as collagen, AA, or thrombin for 5 min. An aliquot (0.35 mL) of the washed rabbit platelets was mixed with 5 mM EDTA on ice, and the mixture was then centrifuged at 12,000 rpm for 2 min. The supernatant was mixed with 6 M trichloroacetic acid (TCA) and centrifuged at 12,000 rpm for 2 min. An aliquot (0.3 mL) of the TCA supernatant was mixed with 1.2 mL 0.5% *O*-phthalaldehyde in a 1:10 ethanol:8N HCl solution, placed in a boiling water bath for 10 min, and then cooled on ice. Excess lipids were extracted using CHCl_3_, and the concentration of TCA was obtained at excitation and emission wavelengths of 360 nm and 475 nm, respectively. The extent of serotonin release was calculated by using a serotonin creatinine sulfate as standard solution.

### Arachidonic Acid Liberation Assay

Arachidonic acid liberation was measured as described previously (Lee et al., 2009). In brief, PRP was preincubated with [^3^H]-AA (1 μCi/mL) at 37°C for 1.5 h and then washed with Hepes buffer 1 (pH 6.5) by centrifugation at 3,000 rpm for 10 min. [^3^H]-AA-labeled platelets (4 × 10^8^ cells/mL) were pretreated with 100 μM BW755C, a COX-1 and LOX inhibitor, and various concentrations of **3** at 37°C for 3 min in the presence of 1 mM CaCl_2_ followed by collagen treatment. The reaction was terminated by the addition of chloroform/methanol/HCl (200/200/1, v/v/v). Lipids were extracted and separated by thin layer chromatography (TLC) plate on silica gel G plates using a petroleum ether/diethyl ether/acetic acid (40/40/1, v/v/v) development system. The areas corresponding to each of the lipids were scraped, and the amount of radio-labeled metabolite was determined by liquid scintillation counting.

### Measurement of Arachidonic Acid Metabolites

TXB_2_, PGD_2_, and 12-HETE generation was measured as described previously (Lee et al., 2014). In brief, washed rabbit platelets (4 × 10^8^ cells/mL) were preincubated with various concentrations of **3** at 37°C for 3 min and further incubated with a mixture of [^3^H]-AA (1 μCi/mL) and unlabeled AA (3 μM) for 5 min. The reaction was terminated by the addition of stop solution (2.6 mM EGTA and 130 μM BW755C). Lipids were extracted and separated by TLC on silica gel G plates using an ethyl acetate/isooctane/acetic acid/H_2_O (117/65/26/130, v/v/v/v) development system. After lipids separation, the radio activities of AA metabolites were measured in the same way described in AA liberation assay section.

### Western Blot Analysis

Total platelet proteins in the Laemmli sample buffer were boiled for 5 min and resolved by 7.5% sodium dodecyl sulfate polyacrylamide gel electrophoresis (SDS-PAGE). Proteins were transferred electrophoretically to a polyvinylidene fluoride (PVDF) membrane (ATTO Corp., Tokyo, Japan) for 80 min at 120 mA. After blocking with TBS-T (10 mM Tris, 150 mM NaCl, 0.1% Tween-20, pH 7.6) containing 5% BSA for 1 h, the membranes were then incubated with a 1:1000 dilution of primary antibodies targeting the following; phospho-extracellular signal-regulated receptor kinase 1/2 (ERK1/2), phospho-protein kinase B (Akt), phospho-PLCγ2, phospho-Syk, phospho-linker for activation of T cells (LAT), phospho-glycogen synthase kinase 3β (GSK3β), ERK1/2, Akt, and PLCγ2 (all from Cell Signaling Technology, Inc.). The primary antibody was removed, and the blots were washed three times in TBS-T. Blots were then incubated with anti-rabbit antibody (AbFrontier, Seoul, South Korea) diluted 1:2000 in TBS containing 5% BSA for 5 h at 4°C and then washed five times in TBS-T. Antibody-bound proteins were detected using enhanced chemiluminescence (AbFrontier, Seoul, South Korea) and imaging systems (Bio-Rad, Hercules, CA, United States) according to the manufacturer’s instructions. The expression level of each protein was normalized to β-actin and the intensities of the bands were quantified using Quantity One (Bio-Rad) (Han et al., 2015a).

### Immunofluorescence Assays

Washed rabbit platelets in CaCl_2_ (1 mM) were incubated for 3 min with **3** (10, 50, and 100 μM) or DMSO and then stimulated with collagen (5 μg/mL) for 5 min. The platelets were seeded on collagen-coated 24-well plates and then incubated at room temperature. After an 1 h incubation, unbound platelets were removed, and adherent platelets were fixed by 3% paraformaldehyde. Fixed cells were stained with FITC anti-GPVI antibody and visualized using confocal fluorescent microscopy.

### Tail Bleeding Assay

Male ICR mice (5 week-old) were purchased from Sam-Tako Animals (Osan, Sout Korea). Mice were allowed to acclimate for 1 week at 24°C with 55% humidity and to have free access to a standard commercial pellet (Wonju, South Korea) and drinking water. All animal handling performed was approved by the Committee (CNU-00731). Tail bleeding model analysis was performed as described previously (Liu et al., 2012). Six-week-old mice were anesthetized by intraperitoneal injection of pentobarbital (50 mg/kg). The anesthetized mice were intravenously (i.v.) administered with vehicle (DMSO/normal saline ratio 1:39), **3** (8.7 and 34.6 mg/kg), and aspirin (ASP, 10 and 20 mg/kg) as a positive control. After 20 min of drug injection, the distal 5 μm of the tail was amputated and immersed in 37°C saline to measure the blood stopping time for 20 min. Body weight was measured before and after the bleeding experiment. The volume of blood loss during the experimental period was calculated based on weight loss.

### Intravital Microscopy

Intravital microscopy analysis was performed as described previously (Morrell et al., 2008). Mice were anesthetized and dosing protocols were the same way as described in tail bleeding assay section. Platelets were labeled with 0.1 mL of rhodamine 6G (0.1%) injected intravenously. The mesentery was externalized, thrombosis was initiated by the addition of a 5-mm^2^ piece of Whatman’s paper soaked with 10% FeCl_3_, and thrombosis was recorded for 3 min using a digital imaging camera and analyzed using software (K1-FluoRT, Nanoscope Systems, South Korea).

### Statistical Analysis

All data are expressed as means ± SEM of three independent experiments. Analysis of variance (ANOVA) was used to compare parameters among multiple groups (GraphPad, San Diego, CA, United States). If a significant difference between treated groups was found, Dunnett’s test was applied. Differences with *p* < 0.05 were considered statistically significant.

## Results

### Effect of Crude Extracts and Constituted Compounds from *L. obtusiloba* on Collagen-Induced Platelet Aggregation

**FIGURE 1:**
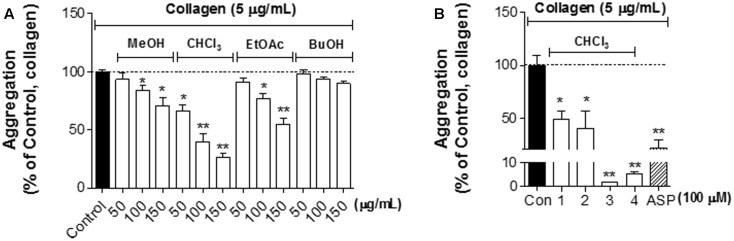
The effect of crude extracts and constituted compounds from *Lindera obtusiloba* on collagen-induced platelet aggregation. **(A,B)** Washed rabbit platelets in CaCl_2_ (1 mM) were pre-incubated for 3 min with *L. obtusiloba* extracts (MeOH, CHCl_3_, EtOAc, and BuOH) (50–150 mg/mL) **(A)** or isolated compounds (**1**–**4**, 100 μM) and aspirin (ASP) **(B)**. Then the cells were stimulated with collagen (5 μg/ml) for 5 min and platelet aggregation was assayed. Data are expressed as the means ± SEM (*n* = 3). ^∗^*p* < 0.05, ^∗∗^*p* < 0.01 vs. control (Con, collagen treatment, but no experimental materials).

To evaluate a beneficial effect of crude extracts and isolated compounds of *L. obtusiloba* on platelet function, washed rabbit platelets were pretreated with collagen followed by treatment of the crude extracts including MeOH, CHCl_3_, EtOAc, and BuOH at 50∼150 μg/mL of concentration and each compound at 100 μM, respectively (**Figures 1A,B**). As shown in **Figure 1A**, each extract of MeOH, CHCl_3_, and EtOAc appeared effective in inhibiting collagen-induced platelet aggregation in a concentration-dependent manner. The CHCl_3_ extract exhibited the highest platelet aggregation inhibitory activity, whereas the BuOH extract did not show any effects. In addition, each isolated compound from CHCl_3_ extract of *L. obtusiloba*, including **1**, **2**, **3**, and **4** significantly inhibited collagen-induced platelet aggregation. Especially, **3** and **4** showed a pronounced inhibitory effect on platelet aggregation compared to the same concentration of ASP, which is a well-known antiplatelet agent (**Figure 1B**) (Zhou et al., 2011). Since **3** showed the most potent inhibitory effect on platelet aggregation among the crude extracts and compounds, we decided to evaluate the role of **3** on the platelet functions.

### Effects of **3** on Platelet Aggregation and Serotonin Secretion

**FIGURE 2:**
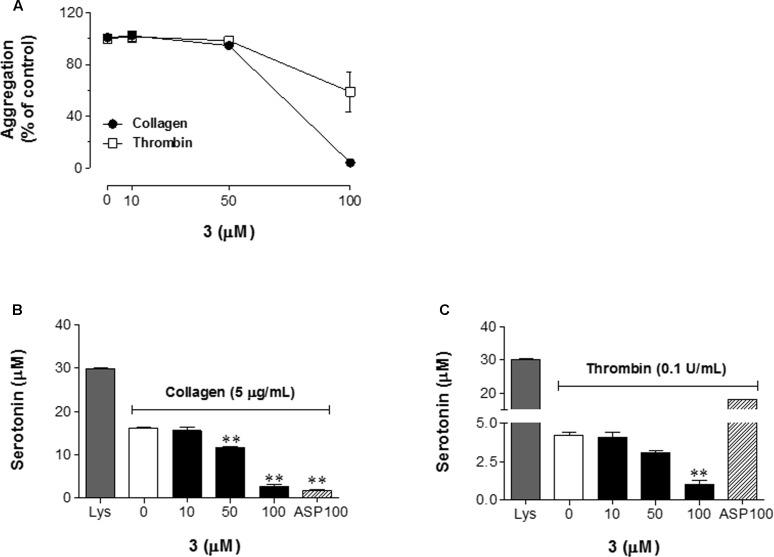
**3** increases platelet aggregation and serotonin secretion. **(A)** Platelet aggregation was induced by the addition of collagen (5 μg/mL), or thrombin (0.1 U/mL). **(B,C)** Washed rabbit platelet suspensions were incubated with a serotonin re-uptake inhibitor, imipramine (5 μM) following **3** (10–100 μM) treatment for 5 min prior to the addition of collagen (5 μg/mL, **B**), or thrombin (0.1 U/mL, **C**). Serotonin secretion in the supernatant was determined by fluorometric assay. Lys indicates total serotonin content in the supernatant of cell lysate. Data are expressed as the means ± SEM (*n* = 3). ^∗∗^*p* < 0.01 vs. control (collagen or thrombin treatment, but no **3**).

Prior to examine the selectivity of **3** on various agonist-induced forms of platelet aggregation, we screened effective concentration of each agonist and decided to utilize 5 μg/mL collagen, and 0.1 U/mL thrombin based on the previous report and our preliminary experiment (Yu et al., 2012). **3** significantly inhibited collagen-induced platelet aggregation, but had a partial effect on thrombin-induced platelet aggregation (**Figure 2A**). To confirm the effect of **3** on serotonin secretion, we measured the amount of serotonin release from supernatants of various agonist-treated platelets using serotonin fluorophore as shown in **Figure 2B** (collagen), and **Figure 2C** (thrombin). **3** treatment reduced collagen-induced serotonin release in a concentration-dependent manner compared to collagen untreated cells. Expressed as a percentage of the control (agonist treatment, but no **3**), collagen-induced serotonin release was 28.13 and 82.68% (**Figure 2B**), and thrombin-induced serotonin release was 27.58 and 75.01% (**Figure 2C**) at 50 and 100 μM of **3**, respectively. These results suggest that **3** exerts a strong inhibitory effect on collagen-induced serotonin release after platelet activation leading to platelet aggregation.

### **3** Inhibits PLCγ2-Akt-ERK1/2-p47 Signaling in Platelets

**FIGURE 3:**
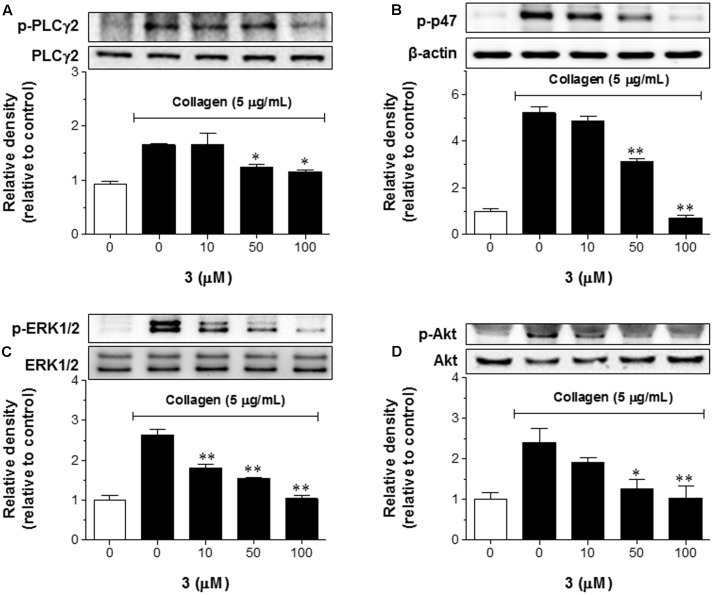
**3** inhibits PLCγ2-mediated signaling pathway. **(A–D)** Washed rabbit platelets in CaCl_2_ (1 mM) were incubated with **3** (10–100 μM) or DMSO for 3 min followed by collagen (5 μg/mL) stimulation for 5 min. Cells were lysed and proteins were employed by SDS-PAGE following Western blotting using primary antibodies targeting anti-phospho PCLγ2 **(A)**, anti-phospho p47 **(B)**, anti-phospho ERK1/2 **(C)**, and anti-phospho Akt **(D)**. The data shown in bar graphs expressed as the means ± SEM are an average of three similar and independent experiments and the gel images are of representative blots. ^∗^*p* < 0.05, ^∗∗^*p* < 0.01 vs. control (collagen treatment, but no **3**).

To understand whether **3** regulates collagen-mediated tyrosine kinase signaling pathway to inhibit platelet activation, phosphorylations of PLCγ2, p47, ERK1/2, and Akt were evaluated by Western blotting (**Figure 3**). When the platelets were pretreated with **3** for 3 min followed by stimulation with collagen, the phosphorylation of PLCγ2 was significantly reduced in **3**-treated platelets (**Figure 3A**). In addition, the phosphorylation levels of p47 (**Figure 3B**) and ERK1/2 (**Figure 3C**) were also reduced. These proteins are also important components of the protein kinase C (PKC) aggregation signaling pathway (Adam et al., 2008). To determine the effect of **3** on the PI3K aggregation signaling pathway, Akt phosphorylation was examined. In a similar manner to the PLCγ2 signaling pathway, **3** treatment significantly inhibited the phosphorylation of Akt in platelets (**Figure 3D**). Together, these data indicate that **3** suppresses platelet activation via inhibition of PLCγ2, p47, ERK1/2, and Akt phosphorylation.

### Effect of **3** on the Liberation of AA and Diacylglycerol and the Formation of TXB_2_ and PGD_2_

**FIGURE 4:**
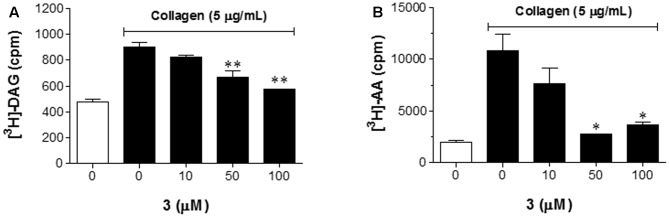
**3** suppresses the production of DAG and the liberation of AA. DAG **(A)** and AA **(B)** liberation assays were performed using [^3^H]-AA-labeled platelets. After platelet suspensions were incubated with **3** (10, 50, and 100 μM) or with a PLC inhibitor CaCl_2_ (1 mM) in the presence of 100 μM indomethacin, the platelets were stimulated with 5 μg/mL collagen for 2 min. The data shown in bar graphs expressed as the means ± SEM are an average of three similar and independent experiments. ^∗^*p* < 0.05, ^∗∗^*p* < 0.01 vs. control (collagen treatment, but no **3**).

**FIGURE 5:**
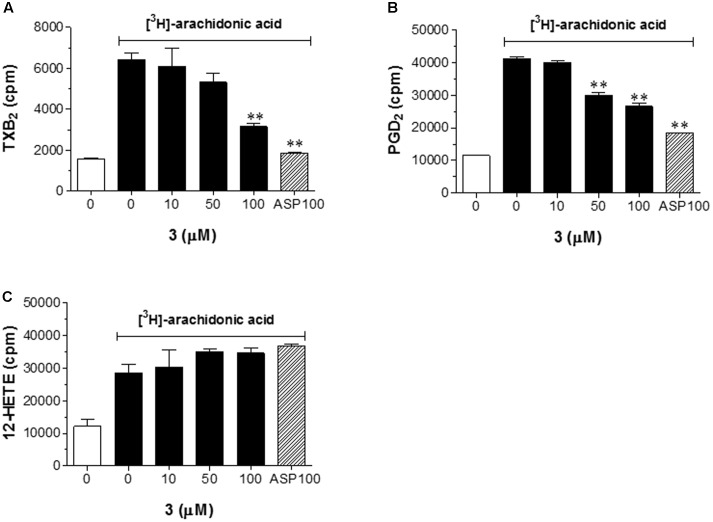
**3** suppresses the formation of TXB_2_ and PGD_2_. AA-mediated formation of [^3^H]-TXB_2_ **(A)**, [^3^H]-PGD_2_ **(B)**, or [^3^H]-12-HETE **(C)** was assayed. [^3^H]-TXB_2_, [^3^H]-PGD_2_, and [^3^H]-12-HETE were extracted and separated by TLC on silica gel G plates. The area corresponding to each lipid was scraped off, and radioactivity was measured by liquid scintillation counting. The results shown in bar graphs expressed as the means ± SEM are an average of three similar and independent experiments. ^∗∗^*p* < 0.01 vs. control ([^3^H]-AA treatment, but no **3**).

To gain insight as to how **3** regulates platelet aggregation, we examined whether **3** is important for collagen-induced production of diacylglycerol (DAG), a known downstream signaling molecule of PLCγ2 phosphorylation, and AA liberation, induced by Ca^2+^ mobilization via inositol-1,4,5-triphosphate (IP_3_) (Lee et al., 2014). DAG and AA levels were measured using [^3^H]-AA-labeled platelets stimulated with collagen (**Figure 4**). Treatment with **3** (50 and 100 μM) significantly inhibited the collagen-induced production of DAG by 55.2 and 76.9%, respectively (**Figure 4A**), indicating that the decrease in DAG production is likely due to the inhibition of PLCγ2 phosphorylation by **3**. In addition, 50 and 100 μM **3** decreased AA release by 90.7 and 80.9%, respectively (**Figure 4B**). The metabolism of AA in activated platelets results in the production of numerous metabolites that play a role in platelet aggregation; thus, we assessed the effect of **3** on the AA-mediated production of TXB_2_ (an inactive metabolite and product of TXA_2_), PGD_2_, and 12-HETE_._ Interestingly, **3** suppressed AA-mediated formation of TXB_2_ (**Figure 5A**) and PGD_2_ (**Figure 5B**) in a dose-dependent manner. However, **3** did not affect AA-mediated 12-HETE production, a known LOX metabolite (**Figure 5C**). Since the production of TXB_2_ and PGD_2_ from AA is mediated by COX pathway (Kuehl and Egan, 1980), these results suggest that **3** could inhibit COX activity rather than LOX.

### **3** Exerts an Anti-aggregation Effect by Inhibiting the Activity of Platelet GPVI Receptor

**FIGURE 6:**
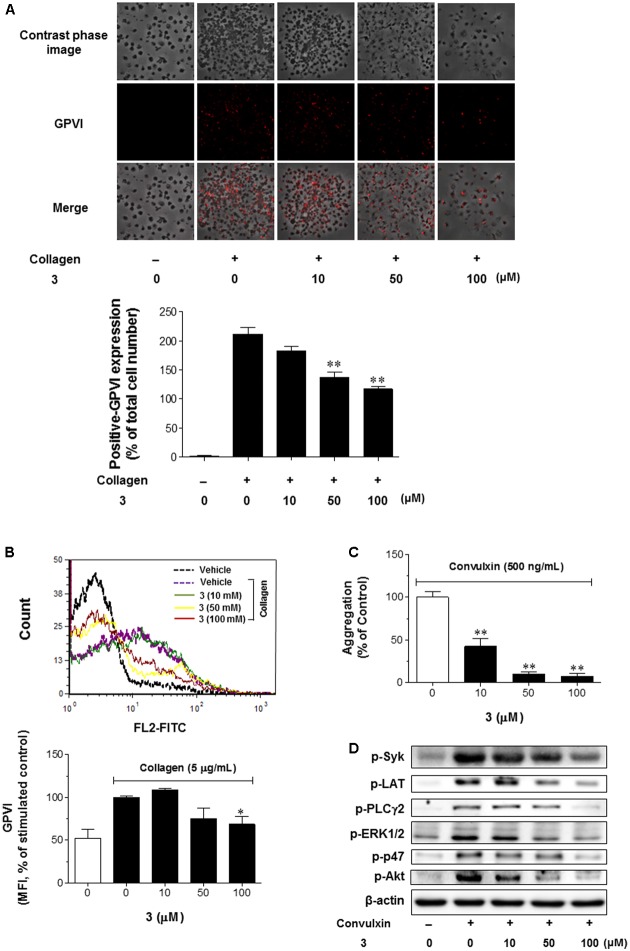
**3** suppresses the activation of platelet receptor GPVI to inhibit platelet aggregation. **(A)** Washed rabbit platelets in CaCl_2_ (1 mM) were incubated for 3 min with **3** (10, 50, and 100 μM) or DMSO and then stimulated with collagen (5 μg/ml) for 5 min. After rinsing the cells with PBS, platelets were added on the collagen-coated 24-well plates. After 1 h incubation at room temperature, unbound platelets were removed, and adherent platelets were fixed by 3% paraformaldehyde and stained for anti-GPVI-FITC antibody. After GPVI staining, platelets were visualized by confocal fluorescent microscopy. The data shown in bar graphs expressed as the means ± SEM represent the GPVI-positive cell numbers after normalization with total cell numbers, and are an average of three similar and independent experiments. The images are representative microscopic pictures from three similar and independent experiments. ^∗∗^*p* < 0.01 vs. control (collagen treatment, but no **3**). **(B)** At the separate experiment set with the same condition, GPVI-FITC positive cells were counted using flow cytometer (upper panel). Bar graph represents the geometric mean percentage of GPVI-positive cells in the non-treated control and collagen-stimulated platelets (lower panel), which are an average of three similar and independent experiments. ^∗^*p* < 0.05 vs. control (collagen treatment, but no **3**). Platelet aggregation **(C)** or intracellular signaling pathway **(D)** was induced by the addition of a GPVI specific agonist convulxin (500 ng/mL). Washed rabbit platelets in CaCl_2_ (1 mM) were incubated for 3 min with **3** (10–100 μM) or DMSO followed by convulxin stimulation for 5 min. Then, platelet aggregation was measured and the results expressed as the means ± SEM are an average of three similar and independent experiments, ^∗∗^*p* < 0.01 vs. control (convulxin treatment, but no **3**) **(C)**. At the separate experiment set with the same condition, total proteins were extracted and western blotting against indicated antibodies was performed to investigate the effect of **3** on GPVI-mediated intracellular signaling pathway **(D)**. The gel images are of representative blots from three similar and independent experiments.

Since GPVI is the predominant collagen receptor on platelets, it mediates collagen-induced platelet aggregation (Inoue et al., 2003). Therefore, we assessed the contribution of GPVI signaling to collagen-induced secretion of platelet soluble factors. First, we used an immunofluorescence assay to determine the GPVI expression under **3** treatment for 3 min following collagen stimulation for 5 min. Platelets were immobilized and fixed to collagen-coated cover plates, and the contrast phase and fluorescent images were taken using a confocal microscopy. When the contrast phase and fluorescent images were merged and examined, it was clear that **3** treatment reduced the level of GPVI expression in a concentration-dependent manner (**Figure 6A**). Platelets treated with **3** (100 μM) inhibits collagen-induced GPVI expression by 52% compared to control (no treatment of **3**, bar graph in **Figure 6A**). To quantitatively validate these images, the effect of **3** on GPVI expression was examined using fluorescence-activated cell sorter. Consistent with results shown in **Figure 6A**, platelets expressed GPVI-FITC were significantly reduced by 100 μM **3** treatment (**Figure 6B**), suggesting that **3** possesses a regulatory effect on GPVI expression in collagen-stimulated platelets. We further investigated whether **3** regulates platelet aggregation via GPVI-mediated signaling pathway. As shown in **Figure 6C**, GPVI agonist convulxin (500 ng/mL)-induced platelet aggregation was significantly inhibited by **3** in a concentration-dependent manner. Convulxin-induced activation of Syk and its downstream signaling molecules LAT and PLCγ2 were significantly reduced by **3** (**Figure 6D** 1st, 2nd, and 3rd panels). Consistent with data shown in **Figure 3**, **3** also inhibited convulxin-induced activation of ERK1/2, p47, and Akt (**Figure 6D** 4th, 5th, and 6th panels). Together, these data clearly indicate that **3** inhibits platelet aggregation through the inhibition of GPVI receptor-mediated signaling pathways.

### **3** Blocks Platelet Aggregation and Delays Thrombosis *In Vivo*

**FIGURE 7:**
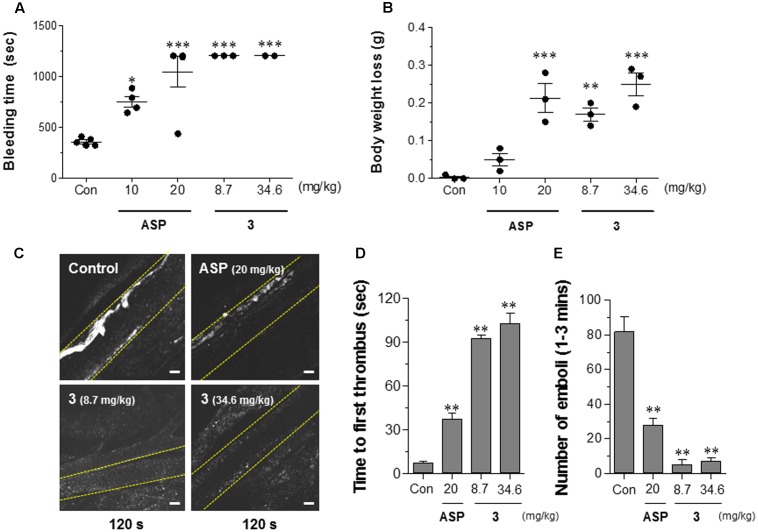
**3** prolongs bleeding time and inhibits thrombus formation through inhibition of platelet aggregation *in vivo*. **(A)** Mean time to cessation of bleeding in tail bleeding assay was performed in control-saline, **3** (8.7 and 34.6 mg/kg), or ASP (10 and 20 mg/kg) treated mice as described in Methods (time < 1200 s, *n* = 3–5 mice in each group). The data expressed as the means ± SEM, ^∗^*p* < 0.05, ^∗∗∗^*p* < 0.001 vs. control (Con, no treatment). **(B)** Body weight was measured right before and after the bleeding test. The results expressed as the means ± SEM, ^∗∗^*p* < 0.01 vs. Con. **(C)** FeCl_3_ was placed on externalized mesenteric arterioles, and platelets were imaged using intravital microscopy (representative images120 s after FeCl_3_). Bars, 100 μm. **(D)** Treatment with 8.7 and 34.6 mg/kg **3** prolongs thrombus formation *in vivo*. Average time required to form a first thrombus of more than 20 μm in diameter. **(E)** Number of emboli with a diameter of more than 20 μm forming 1–3 min after FeCl_3_ injury. The results expressed as the means ± SEM, ^∗∗^*p* < 0.01 vs. Con.

To assess the effect of **3** on platelet function, the delayed time in tail bleeding was examined *in vivo*. ASP, which has an antiplatelet action, was used as a positive control. In order to determine the effective concentration of ASP and 3 in mouse, equivalent concentrations were applied based on the concentration of ASP used in clinical test (Nair and Jacob, 2016). The experiments were conducted at low and high concentrations to determine the effects of **3** on organ toxicity as well as platelet function. The mice were treated with saline (control), **3** (8.7 and 34.6 mg/kg), or ASP (10 or 20 mg/kg), and 20 min later, tail bleeding times were determined. Control mice have a mean bleeding time of ∼320 s (**Figure 7A**). Treatment with low and high concentrations of ASP showed delayed mean bleeding time to 750 and 1044 s, respectively. Interestingly, mice treated with **3** at both low and high concentrations exhibited prolonged mean bleeding time (over 1200 s) (**Figure 7A** and Supplementary Figure 4) and significant increase in blood loss based on change in body weight compared to control and ASP treated mice (**Figure 7B**). To confirm these data, we examined the effect of **3** on thrombus formation using intravital microscopy. Mice were treated with saline (control), **3**, and ASP intravenously 20 min before mesentery externalization and 10% FeCl_3_ application. Platelets were labeled by intravenous injection of 0.1 mL of rhodamine 6G (0.1%). Images were collected to quantify platelet function *in vivo* (**Figure 7C**). Compared with saline-treated control group, mice treated with ASP or **3** have prolonged time to thrombus formation (first stable thrombus > 20 μm) (**Figure 7D**) and show the decreased number of emboli (**Figure 7E**). The organ toxicity of high concentration of agents (34.6 mg/kg **3** and 20 mg/kg ASP) was evaluated in different organs including heart, lungs, liver, spleen, and kidney using apoptosis markers, such as cleavage of poly (ADP-ribose) polymerase (PARP) and caspase-3 (Supplementary Figure 5). Note that **3** and ASP administration during tail bleeding and thrombus evaluation at high concentration did not show any cleaved forms of PARP and caspase 3 compared to control group, which indicate no organ toxicity at high concentration of agents used in this study.

## Discussion

This study has two major findings: (1) **3** isolated from *L. obtusiloba* extract exhibited strong inhibitory effects on collagen-induced platelet aggregation and activation, and (2) the antiplatelet action of **3** occurs via inhibiting thrombotic metabolite formation and GPVI-mediated signaling pathway. This is the first report that **3** derived from *L. obtusiloba* extract acts on collagen receptor-mediated signal transduction mechanism and blocks the formation of COX-mediated products associated with platelet aggregation, leading to antiplatelet action. Our observations suggest that **3** may be a new therapeutic candidate for the prevention/treatment of atherosclerosis and vascular restenosis.

The ethanol extracts of *L. obtusiloba* have shown strong antioxidant activities, which contribute to liver protection against *t-BHP* (tert-butyl hydroperoxide)-induced toxicity both *in vitro* and *in vivo* (Hong et al., 2013b). It has been reported that *L. obtusiloba* included bioactive compounds such as neolignans, lignans, phenolic glycosides, flavonol glycosides, and butanolides (Choi et al., 2013, 2014; Hong et al., 2013a). The antioxidant and other bioactive properties of lignans, quercitrin, and phenolic glycosides in natural plants have been extensively studied in various cell types and tissues (Choi et al., 2014). There are only few reports for butanolides from natural plants to have an inhibitory effect on ADP-induced platelet aggregation (Dong et al., 2013). However, there is still the lack of information about their underlying mechanisms and confirming their bioactivities using animal models. Our data demonstrated that CHCl_3_ extract appeared the strongest inhibitory effect on collagen-induced platelet aggregation compared to other extracts (**Figure 1A**). The CHCl_3_ extract included one lignin (**1)** and three butanolides (**2**, **3**, and **4)** (**Figure 1B**). **1** has been isolated from the bark of *Fagara* plants and from sesame oil and showed various effects on cholesterol lowering (Liang et al., 2015), antioxidant, anti-cancer (Xu et al., 2015), and anti-proliferative in vascular smooth muscle cells (Han et al., 2015b). Although **1** showed the inhibitory effect on platelet aggregation by 48.2%, butanolide compounds, **3** and **4** inhibited collagen-induced platelet aggregation by more than 90% (**Figure 1B**). Compound **4** has a larger number of carbon than compounds **2** and **3** which have the same number. Interestingly, the antiplatelet action of compound **3** is greater than that of compound **2** in which there is a carbon-carbon double bond at the end. It suggests that C-C double bond in structure and the number of carbon may affect antiplatelet action of butanolides.

During the progression of platelet activation, AA is converted to various metabolites that are closely involved in these bidirectional modulations, especially TXA_2_ and PGD_2_, which are produced by COX pathway, and 12-HETE generated via the LOX pathway (Chen et al., 1992). Especially, TXA_2_ and PGD_2_ are potent vasoconstrictors and inducers of platelet aggregation (Sekiya et al., 1991). In our current study, we found that **3** also inhibited the conversion of AA to TXA_2_ and PGD_2_ but not 12-HETE (**Figure 5**), which suggests that it possesses significant inhibitory activity on COX pathway.

The process of atherothrombosis is initiated from endothelial damage-mediated collagen exposure to the circulating platelets (Badimon et al., 2009). The circulating platelets bind directly to collagen with collagen-specific GPVI receptor to trigger a signaling pathway, which results in activation of platelet integrins (Nieswandt and Watson, 2003). Our present data suggested that pretreatment of platelets with **3** abolished GPVI expression on the cell surface and led to down-regulation of GPVI-mediated signaling pathway (**Figure 6**). It has been reported that phosphorylation of Syk and PLCγ2-mediated downstream signaling pathway were involved in GPVI activation responded by collagen (Berlanga et al., 2000). In fact, **Figure 6** showed that **3** inhibited collagen-induced platelet aggregation and GPVI agonist (convulxin)-activated Syk-PLCγ2 and downstream events, ERK1/2, p47, and Akt were inhibited by **3** in the platelets. These results clearly indicate that **3** selectively inhibits platelet aggregation by inhibiting Syk-PLCγ2 signaling pathway and COX-mediated formation of platelet aggregatory products such as TXA_2_ via GPVI.

Consistent with *in vitro* experiments showing the inhibitory effect of **3** on platelet aggregation, we observed significant prolongation of *in vivo* tail bleeding time and blood loss in **3**-treated mice compared to control group (**Figures 7A,B** and Supplementary Figure 2). Moreover, FeCl_3_-induced arterial thrombosis was significantly delayed in three-treated mice compared to saline-treated control group, suggesting the strong effect of **3** on platelet aggregation (**Figures 7C–E**).

In conclusion, this study demonstrated that the **3**, which is one of the butanolides fractionated from *L. obtusiloba* extract, has significant regulatory effect on collagen-induced platelet activation and aggregation through inhibiting aggregatory metabolite formation and GPVI signaling pathway. Therefore, our observations suggest that the beneficial effect of **3** on thrombotic disease might be due its ability to modulate platelet activity.

## Author Contributions

S-HJ performed most of the experiments and prepared the manuscript. J-HH and H-SP contributed to experiments. J-JL gave the concept of the work. SY and YK performed isolation of compounds and structure elucidation. K-SH revised the manuscript. C-SM were the group leaders from two independent groups offering supervision and financial support. All authors read and approved the final manuscript.

## Conflict of Interest Statement

The authors declare that the research was conducted in the absence of any commercial or financial relationships that could be construed as a potential conflict of interest.
