# Supplementary material for: Inhibition of Collagen-Induced Platelet Aggregation by the Secobutanolide Secolincomolide A from Lindera obtusiloba Blume
Source: Front Pharmacol. 2017 Aug 22;8:560. doi: 10.3389/fphar.2017.00560 (PMC5572288; doi:10.3389/fphar.2017.00560)
Supplement: Supplementary file 1 [file Presentation_1.pdf]

Inhibition of Collagen-Induced Platelet Aggregation by the Butanolide Secolincomolide A from *Lindera obtusiloba* Blume

SUPPLEMENTARY INFORMATION

Extraction and isolation. The air-dried stems of *Lindera obtusiloba* Blume (3 kg) was finely sliced and extracted with methanol (MeOH) to 30 times under refluxing. Concentrated MeOH extract (185.3g, 6.1%) was suspended in H_2O and partitioned with chloroform ($CHCl_3$), ethyl acetate (EtOAc), butanol (BuOH), and water (H_2O), respectively. The $CHCl_3$ extract (52.4 g) was subjected to silica gel column chromatography with a gradient of *n*-hexane-EtOAc (3:1-1:1) to give 3A fraction. Fraction 3A was further chromatographed on a silica gel column using a gradient of *n*-hexane-EtOAc (8:1-7:1) to give 35D fraction and then chromatographed on a silica gel column with $CHCl_3$-MeOH (90:1-70:1) to obtain five subfractions (36A-36E). Subfraction 36A was further purified to silica gel column chromatography with a gradient of $CHCl_3$-EtOAc (60:1) to get compound 1 (124.9 mg). Subfraction 36B was chromatographed on a silica gel column with *n*-hexane-Acetone (8:1) to obtain four subfractions (42A-42D), further purification of the subfraction 42D led to compounds 2 (49.9 mg). Subfraction 42A and 47B was further reverse-phase (RP) chromatography column with *MeOH-Acetone-H2O (3:4:1.2)* to obtain compounds 3 (34.1 mg) and 4 (22.4 mg). Their structures were elucidated utilizing spectrometric and spectroscopic approaches and relevant reported data.

Asarinin, $C_{20}H_{18}O_6$; $[\alpha]_D^{25}$ +131.31 (c 0.1, $CHCl_3$); HR-ESI-MS *m/z* 377.0894 $[M+Na]^+$ (calcd. for $C_{20}H_{18}O_6Na$: 377.1001) ; IR (transmission) ν_{MAX} 2962, 2930, 2867, 1503, 1487, 1438, 1375, 1253, 1179 cm^{-1}. ^1H-NMR (400 MHz, $CDCl_3$) δ: 6.87 (2H, d, J = 2.7 Hz, H-

2, 2'), 6.82 (2H, d, *J* = 7.9 Hz, H- 5, 5'), 6.79 (2H, dd, *J* = 7.9, 2.7 Hz, H-6, 6'), 5.97 (2H, s, -OCH_2O-), 5.95 (2H, s, -OCH_2O-), 4.83 (1H, d, J = 6.5 Hz, H-7), 4.39 (1H, d, *J* = 6.5 Hz, H-7'), 4.10 (1H, d, *J* = 8.8 Hz, H-9'b), 3.85 (1H, d, *J* = 8.1 Hz, H-9b), 3.82 (1H, d, *J* = 8.8 Hz, H-9'a), 3.32 (1H, m, H- 8), 3.29 (1H, d, *J* = 8.1 Hz, H-9a), 2.87 (1H, m, H-8'). ^{13}C-NMR (100 MHz, $CDCl_3$) δ: 147.9 (C-3'), 147.6 (C-4), 147.2 (C-4'), 146.5 (C-3), 135.1 (C-1'), 132.2 (C-1), 119.6 (C-6'), 118.6 (C-6), 108.1 (C-5', C-5), 106.5 (C-2'), 106.4 (C-2), 101.0 (-OCH_2O-), 100.9 (-OCH_2O-), 87.6 (C-7'), 81.9 (C-7), 70.8 (C-9'), 69.6 (C-9), 54.5 (C-8'), 50.8 (C-8).

Secoisolitsealiicolide B, $C_{18}H_{30}O_4$; $[\alpha]_D^{25}$ -52.3 (c 0.3, $CHCl_3$); HR-ESI-MS *m/z* 333.1964 $[M+Na]^+$ (calcd. for $C_{18}H_{30}O_4Na$: 333.2042); IR (transmission) ν_{MAX} 3401, 1733 cm^{-1}. ^{1}H-NMR (600 MHz, $CDCl_3$) δ: 7.08 (1H, t, *J* = 7.6 Hz, H-6), 5.81 (1H, ddt, *J* = 17.4, 10.5, 6.9 Hz, H-16), 4.99 (1H, dd, *J* = 17.4, 1.7 Hz, H-17a), 4.93 (1H, dd, *J* = 10.5, 1.7 Hz, H-17b), 4.90 (1H, s, H-3), 4.03 (1H, s, 3-OH), 3.73 (3H, s, 1- OCH_3), 2.16 (3H, s, H-5), 2.35 (2H, q, *J* = 7.6 Hz, H-7), 2.04 (2H, q, *J* = 6.9 Hz, H-15), 1.52 (2H, m, H-8), 1.28 (12H, br s, H-9 ~ H-14). ^{13}C-NMR (150 MHz, $CDCl_3$) δ: 206.5 (C-4), 166.6 (C-1), 149.2 (C-6), 139.2 (C-16), 129.8 (C-2), 114.2 (C-17), 73.4 (C-3), 52.0 (1-OCH_3), 33.7(C-15), 29.5 ~ 28.7* (C-9 ~ C-14), 28.8 (C-7), 28.6 (C-8), 24.9 (C-5).

Secolincomolide A, $C_{18}H_{32}O_4$, $[\alpha]_D^{25}$ -13.1 (c 0.3, $CHCl_3$), HR-ESI-MS *m/z*: 335.2121 $[M+Na]^+$ (calcd. for $C_{18}H_{32}O_4Na$: 335.2198); IR (transmission) ν_{MAX} 3457, 1727 cm^{-1}. ^{1}H-NMR (400 MHz, $CDCl_3$) δ: 7.08 (1H, t, *J* = 7.5 Hz, H-6), 4.90 (1H, s, H-3), 4.02 (1H, s, 3-OH), 3.73 (3H, s, 1-OCH3), 2.35 (2H, q, *J* = 7.5 Hz, H-7), 2.15 (3H, s, H-5), 1.51 (2H, m, H-8), 1.25 (16H, br s, H-9 ~ H-16), 0.87 (3H, t, *J* = 6.8 Hz, H-17). ^{13}C-NMR (100 MHz,

$CDCl_3$) δ: 206.5 (C-4), 166.6 (C-1), 149.2 (C-6), 129.7 (C-2), 73.3 (C-3), 52.0 (1-OCH_3), 31.8 ~ 29.3* (C-9 ~ C-16), 28.8 (C-8), 28.6(C-7), 24.7 (C-5), 14.0 (C-17).

Secomahubaolide, $C_{24}H_{44}O_4$; $[\alpha]_D^{25}$ -11.3 (c 0.2, $CHCl_3$); HR-ESI-MS *m/z* 397.2393 $[M+H]^+$ (calcd. for $C_{24}H_{45}O_4$: 397.3318); IR (transmission) ν_{MAX} 3445, 1732 cm^{-1}. 1H-NMR (400 MHz, DMSO- d_6) δ: 6.82 (1H, t, J = 7.2 Hz, H-6), 5.42 (1H, s, 3-OH), 2.26 (2H, q, J = 7.2 Hz, H-7), 2.11 (3H, s, H-5), 4.84 (1H, s, H-3), 3.61 (3H, s, 1-OCH_3), 1.41 (2H, m, H-8), 1.24 (28H, br s, H-9 ~ H-22), 0.85 (3H, t, J = 6.2 Hz, H-21). ^{13}C-NMR (100MHz, DMSO-d_6) δ: 208.9 (C-4), 166.2 (C-1), 146.8 (C-6), 132.0 (C-2), 72.2 (C-3), 51.6 (1-OCH_3), 29.0 ~ 28.7* (C-9 ~ C-22), 28.1 (C-8), 27.9 (C-7), 25.8 (C-5), 13.9 (C-23).

*Overlapped signals.

Western blot analysis. Liver, lung, heart, kidney and spleen of mouse were removed right after animal experiment. The extracted tissues were pulverized through a tissue grinder pestle and homogenized by adding CETi lysis buffer (Translab, Daejeon, Korea). The homogeneous liquid was centrifuged at 13,000 rpm at -4°C to obtain a supernatant (Ishii et al., 2004). The supernatant was boiled for 5 min with the Laemmli sample buffer and resolved by 7.5% sodium dodecyl sulfate polyacrylamide gel electrophoresis (SDS-PAGE). Proteins were transferred electrophoretically to a polyvinylidene fluoride (PVDF) membrane (ATTO Corp., Tokyo, Japan) for 80 min at 120 mA. After blocking with TBS-T (10 mM Tris, 150 mM NaCl, 0.1% Tween-20, pH 7.6) containing 5% bovine serum albumin (BSA) for 1 h, the membranes were then incubated with a 1:1000 dilution of primary antibodies targeting the following; poly (ADP-ribose) polymerase (PARP), caspase-3 and β-actin (all from Cell Signaling Technology, Inc.). The primary antibody was removed, and the blots were washed three times in TBS-T. Blots were then incubated

with anti-rabbit antibody (AbFrontier, Seoul, Korea) diluted 1:2000 in TBS containing 5% BSA for 5 h at 4°C and then washed five times in TBS-T. Antibody-bound proteins were detected using enhanced chemiluminescence (AbFrontier, Seoul, Korea) and imaging systems (Bio-Rad, Hercules, CA, USA) according to the manufacturer's instructions. The expression level of each protein was normalized to β-actin and the intensities of the bands were quantified using Quantity One (Bio-Rad).

FIGURES and FIGURE LEGENDS

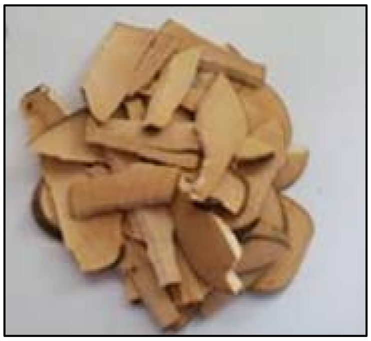

SUPPLEMENTARY FIGURE 1. The stems of *L. obtusiloba*.

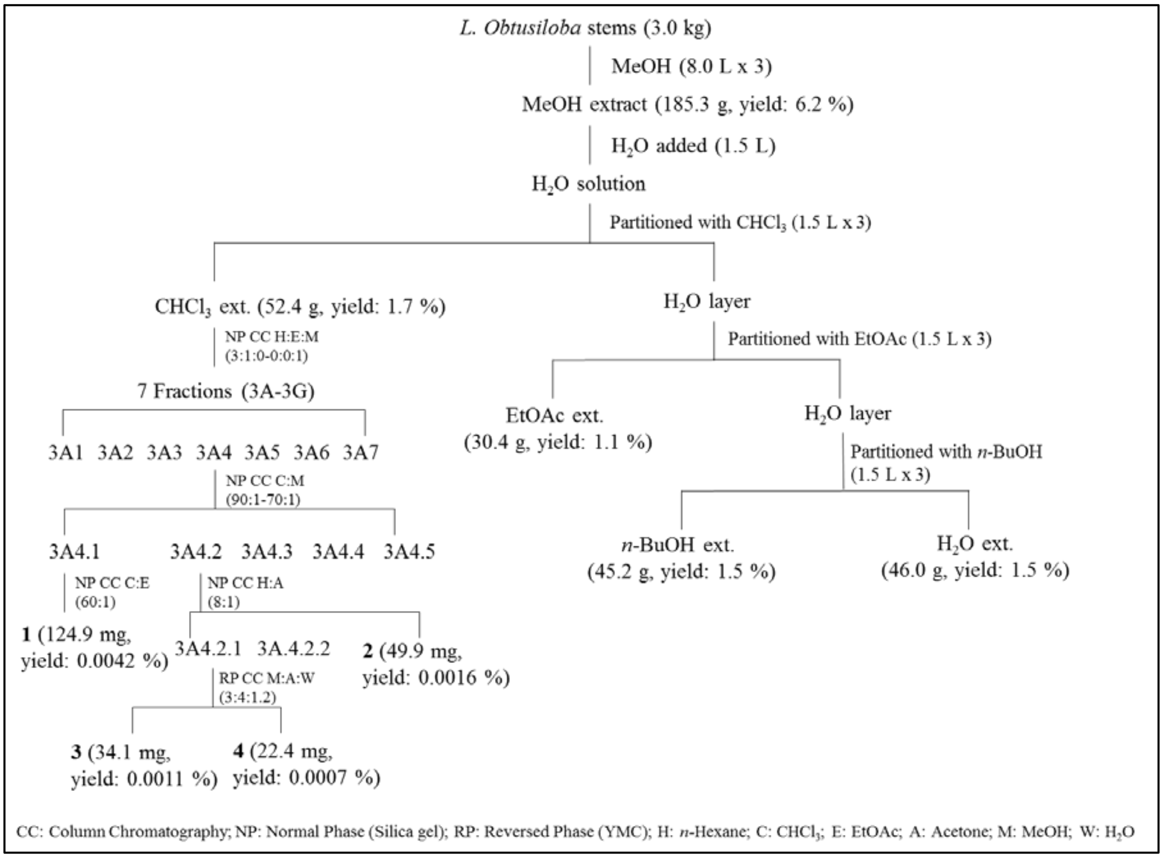

SUPPLEMENTARY FIGURE 2. Isolation scheme of compounds 1-4.

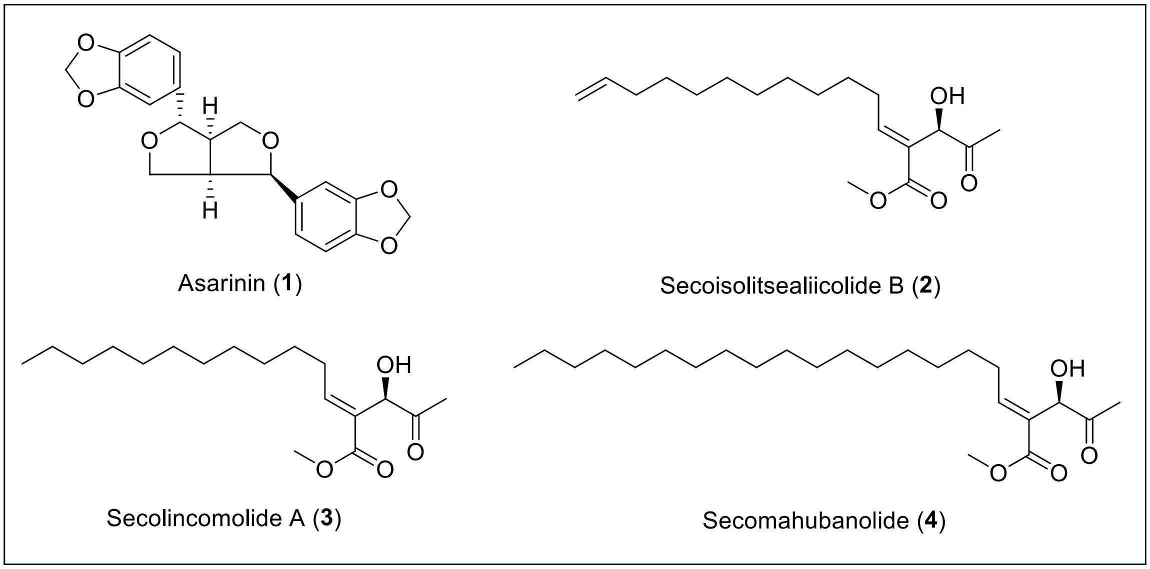

SUPPLEMENTARY FIGURE 3. The chemical structures of isolated compounds from the $CHCl_3$ extracts of *L. obtusiloba*. The structures are elucidated as asarinin (**1**), secoisolitsealiicolide B (**2**), secolincomolide A (**3**), and secomahubanolide (**4**).

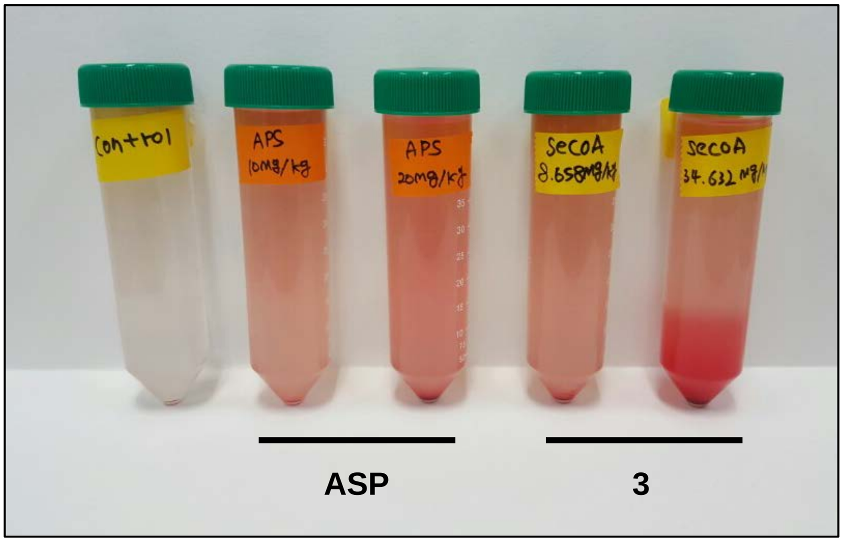

SUPPLEMENTARY FIGURE 4. The effect of secolincomolide A (3) on tail bleeding. Image shows the amount of blood in saline (37°C) after tail bleeding assay in mice treated with control (saline), secolincomolide A (Seco A, 8.7 and 34.6 mg/kg), or aspirin

(ASP, 10 and 20 mg/kg) as described in Materials and Methods section (time < 1200 s, n = 3-5 mice in each group).

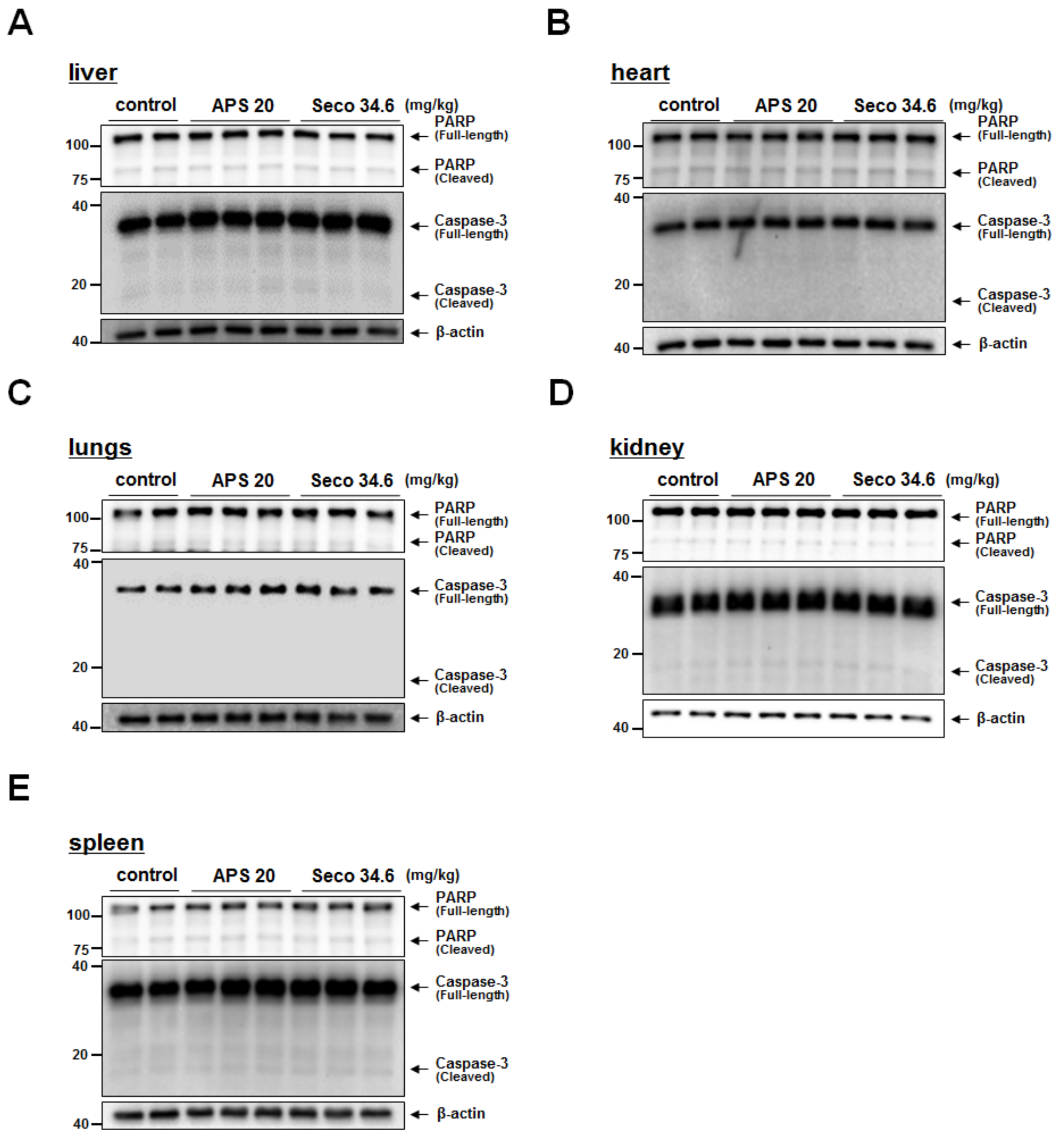

SUPPLEMENTARY FIGURE 5. Evaluation of organ toxicity after administration of high-dose secolincomolide A and aspirin. Tissue lysates were collected from heart, lung, liver, spleen, and kidney of the mice administered with high concentration of

secolincomolide A (Seco) and aspirin (ASP), and dissolved using sodium dodecyl sulfate polyacrylamide gel electrophoresis (SDS-PAGE). Western blotting using primary antibodies targeting anti-poly (ADP-ribose) polymerase (PARP) and –caspase-3 was performed to assess organ toxicity. The expression level of each protein was normalized to β-actin. Control indicates no treatment.

References

Ishii, I., Akahoshi, N., Yu, X.N., Kobayashi, Y., Namekata, K., Komaki, G., et al. (2004). Murine cystathionine γ-lyase: complete cDNA and genomic sequences, promoter activity, tissue distribution and developmental expression. *Biochem. J.* 381(Pt 1)**,** 113-123. doi: 10.1042/BJ20040243
